# Calcineurin Inhibitor–Free, mTOR‐Based Immunosuppression in Chimeric Antigen Receptor T‐Cell Therapy for Post‐Transplant Lymphoproliferative Disorder

**DOI:** 10.1002/jha2.70437

**Published:** 2026-10-28

**Authors:** Maria Mappoura, Yara Hajj Younes, Sisi Wang, Kadiatou Baldet, Carmen De Ramon Ortiz, Sarah Morin, Stavroula Marouridi‐Levrat, Amandine Pradier, Astrid Melotti, Pragallabh Purwar, Chiara Bernardi, Liza Ho, Kristof Egervari, Julie Ducreux, Thomas Longval, Francesco Bertoni, Yves Chalandon, Fadi Haidar, Sophie de Seigneux, Jean Villard, Valérie Olivier, Federico Simonetta

**Affiliations:** ^1^ Division of Hematology Department of Oncology Geneva University Hospitals Geneva Switzerland; ^2^ Translational Research Center For Oncohematology Department of Medicine Faculty of Medicine University of Geneva Geneva Switzerland; ^3^ Department of Pathology and Immunology Faculty of Medicine University of Geneva Geneva Switzerland; ^4^ Nephrology and Hypertension Division Department of Medicine Geneva University Hospitals Geneva Switzerland; ^5^ Division of Clinical Pathology Geneva University Hospital Geneva Switzerland; ^6^ Institute of Oncology Research Faculty of Biomedical Sciences Università Della Svizzera Italiana Bellinzona Switzerland; ^7^ Oncology Institute of Southern Switzerland Ente Ospedaliero Cantonale Bellinzona Switzerland; ^8^ Transplantation Immunology Unit and National Reference Laboratory for Histocompatibility Department of Diagnostic Geneva University Hospitals Geneva Switzerland; ^9^ Geneva Centre for Inflammation Research (GCIR) Geneva Switzerland

**Keywords:** calcineurin inhibitor, chimeric antigen receptor T‐cell (CAR‐T), everolimus, immunosuppression, post‐transplant lymphoproliferative disorder (PTLD), solid organ transplantation, tacrolimus

## Abstract

**Introduction:**

Relapsed/refractory post‐transplant lymphoproliferative disorder (PTLD) remains a challenging complication after solid organ transplantation (SOT), requiring complex balancing of immune‐suppressive therapy (IST) and lymphoma‐directed therapies.

**Methods:**

We report the case of a 40‐year‐old female kidney‐pancreas transplant recipient with relapsing monomorphic PTLD treated with CD19‐chimeric antigen receptor (CAR) T cells therapy (axicabtagene ciloleucel).

**Results:**

Tacrolimus discontinuation and immunosuppression with everolimus and corticosteroids permitted robust CAR T‐cell expansion, complete lymphoma remission, and preservation of graft function. In vitro studies showed that tacrolimus inhibited CAR T‐cell function, while everolimus preserved it, supporting the use of mTOR‐based, calcineurin‐inhibitor‐free IST during CAR T‐cell therapy in SOT recipients.

**Conclusion:**

mTOR‐based, calcineurin inhibitor–free immunosuppression can preserve CAR T‐cell function and enable effective therapy in SOT recipients with PTLD.

## 1 Introduction

Immunodeficiency‐associated lymphoproliferative disorders occurring after transplantation, previously known as post‐transplant lymphoproliferative disorders (PTLDs), are serious complications following solid organ transplantation (SOT) or hematopoietic stem cell transplantation. Treatment of relapsed/refractory PTLD remains difficult and is still associated with poor outcomes [1]. CD19‐CAR T‐cell therapy has revolutionized treatment of aggressive B‐cell lymphomas in immunocompetent patients [2, 3], however its application in SOT recipients remains limited due to concerns regarding efficacy related to ongoing immunosuppressive therapy (IST) and risk of graft rejection. Case reports and small case series have demonstrated the feasibility of CAR T‐cell therapy in SOT recipients with PTLD, with good response rates and acceptable toxicity profiles [4, 5, 6, 7, 8, 9]. However, a major challenge remains the risk of graft rejection, reported in more than 10% of patients [8] and optimal management of baseline immunosuppression remains to be defined. We report here the case of a simultaneous pancreas‐kidney (SPK) recipient with PTLD treated with CAR T cells during calcineurin‐inhibitors (CNI)‐free, mTOR‐based IST. In addition, we provide in vitro evidence that tacrolimus impairs CD19‐CAR T cells antitumor activity while everolimus is permissive to their function.

## 2 Materials and Methods

### 2.1 Cell Lines

Cell lines were cultured in RPMI 1640 medium supplemented with 5% fetal bovine serum (FBS) and 1% penicillin‐streptomycin (Gibco) at 37°C. OCI‐AML‐2 mCherry/luc cells were transduced with a retroviral vector encoding CD19 (MSCV‐hCD19 Addgene Plasmid #127889), and FACS‐sorted for CD19. Cell lines (Farage, SUDHL‐10, DOHH‐2, OCI‐LY‐8, OCI‐AML2‐CD19) expressing mCherry were individually seeded at a concentration of 10^5^ cells/mL in 96‐well clear flat‐bottom plates. Tacrolimus and everolimus (MCE) were diluted in dimethyl sulfoxide (DMSO) and subsequently added to culture media to achieve final concentrations of 5 and 10 ng/mL. Cells treated with 0.1% DMSO were used as control. Tumor growth was monitored using an Incucyte SX5 Live‐Cell Analysis System over 4 days.

### 2.2 Human CD19 CAR T Generation

Human peripheral blood mononuclear cells (PBMCs) were isolated from buffy coats of healthy donors and cryopreserved until use. Frozen PBMCs were thawed and activated for 3 days using Dynabeads Human T‐Activator CD3/CD28 (Gibco) with recombinant human IL‐2 (50 U/mL, PeproTech), in RPMI1640 medium supplemented with 10% heat‐inactivated FBS and 1% penicillin‐streptomycin at 37°C with 5% CO_2_. Activated T cells were transduced using retronectin‐coated (Takara) plates with retroviral supernatant encoding a second‐generation anti‐CD19‐CD28‐CD3ζ CAR construct, generously provided by Dr. Crystal Mackall (Stanford University). Following transduction, Dynabeads were removed, and cells were washed and cultured in complete medium supplemented with 100 U/mL IL‐2 until cryopreservation on Day 14. CAR expression was assessed using APC‐conjugated streptavidin (BioLegend) binding to biotinylated protein L (ThermoFisher). Prior to use, CAR T cells were thawed, washed, and rested for 4 h at 37°C.

### 2.3 Killing Assay

CD19‐CAR T cells and OCI‐AML2‐CD19 mCherry cells were co‐cultured in 384‐well flat‐bottom plates, in triplicates at an effector‐to‐target (E:T) ratio of 2:1, in the presence of tacrolimus or everolimus at 5 and 10 ng/mL or DMSO. Tumor expansion was monitored using the Incucyte SX5 Live‐Cell Analysis System over 7 days.

## 3 Results

### 3.1 Case Presentation

A 40‐year‐old female patient was diagnosed with stage IIE (breast) lymphoma associated with immune deficiency and dysregulation per WHO 2022 classification [10], previously known as monomorphic PTLD, 3 years after SPK transplantation for type 1 diabetes and end‐stage renal disease secondary to diabetic nephropathy while on IST with mycophenolate mofetil (MMF) and tacrolimus. Further histological subclassification was compatible with diffuse large B‐cell lymphoma (DLBCL), activated B‐cell (ABC) subtype. IST was decreased, MMF was discontinued and replaced by low dose corticosteroids. The patient received a first line therapy with Rituximab 375 mg/m^2^ for four weeks achieving complete remission (CR), followed by four courses of rituximab monotherapy every 21 days [11]. Corticosteroids were replaced by everolimus and tacrolimus [12]. Two years later, the disease relapsed, and the patient received a second line of therapy with 4 cycles of rituximab, cyclophosphamide, doxorubicin hydrochloride, vincristine, and prednisone (R‐CHOP) achieving a second CR. In the context of relapse, everolimus was withdrawn and replaced by low dose corticosteroids. Unfortunately, the patient presented a second relapse 2 years afterwards while on prednisone 5 mg/day and tacrolimus. We decided to proceed to CAR T‐cell therapy by axicabtagene ciloleucel in third line. Tacrolimus was discontinued 15 days before leukoapheresis and prednisone increased at 10 mg/day. On the day of apheresis, the patient presented high fever, abdominal pain, weight gain, deterioration of renal function and inflammatory syndrome. Renal biopsy confirmed acute kidney transplant T‐cell mediated rejection stage IIa according to Banff Classification with no feature of antibody‐mediated rejection. No donor‐specific antibodies were detectable. There was no clinical nor biological sign of pancreas rejection. Immediately after leukoapheresis, the patient received high doses of methylprednisolone and IST with tacrolimus and everolimus was re‐initiated leading to kidney function recovery. Rituximab bridging (four weekly doses of 375 mg/m^2^) led to a partial response.

At time of hospital admission for CAR‐T cell infusion, the patient was on IST with prednisone 10 mg/day, tacrolimus and everolimus. Tacrolimus was discontinued and prednisone was increased to 20 mg/day on the first day of lymphodepletion treatment (fludarabine 30 mg/m^2^ and cyclophosphamide 500 mg/m^2^) while everolimus was continued (target levels 5–6 µg/L). Axicabtagene ciloleucel administration was delayed until Day 8 since the beginning of lymphodepletion when tacrolimus was indetectable (< 0.5 µg/L). Patient presented cytokine release syndrome (CRS) grade 1 at Day 4 after CAR‐T administration with no other complications. CAR‐T cell expansion was observed by droplet digital PCR (Figure 1A) and by flow cytometry (Figure 1B). A PET‐CT performed at Day 7 after CAR T‐cell therapy showed a complete metabolic response (Figure 1C) which persisted at 1, 3 and 6 months after CAR T cells. Due to severe proteinuria, everolimus was discontinued three months after CAR T cells and replaced by tacrolimus (target levels 3‐4 ug/L), associated with prednisone 5 mg/day. Unfortunately, the PTLD relapsed 12 months after CAR T cells. Although causality cannot be established, we cannot exclude the possibility that reintroducing tacrolimus contributed to the subsequent relapse by impairing residual CAR T‐cell activity and/or endogenous antitumor immunity.

**FIGURE 1:**
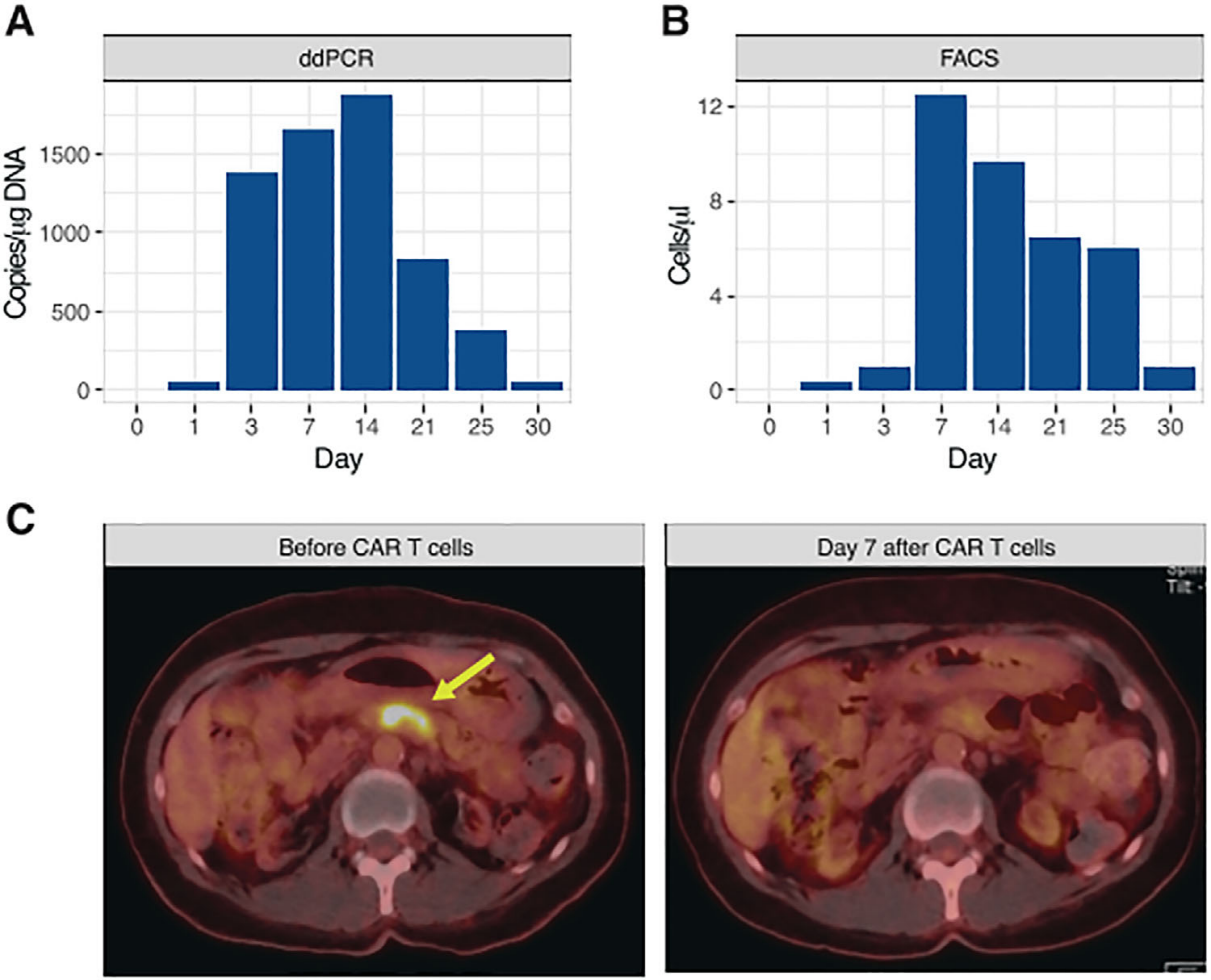
Everolimus‐based, CNI‐free immunosuppression allowed complete response to CAR T cells in a SOT recipient treated for PTLD. (A, B) CAR T‐cell expansion post‐infusion assessed by (A) droplet digital PCR and (B) flow cytometry. (C) PET‐CT scans obtained before and 7 days after CAR T‐cell therapy showing obtention of a complete metabolic remission.

### 3.2 In Vitro Evaluation of the Effect of Tacrolimus and Everolimus on CAR T‐Cell Antitumor Activity

Our decision to withhold tacrolimus while pursuing everolimus treatment was based on the hypothesis that calcineurin inhibition would preclude, while mTOR inhibition would allow, CAR T‐cell antitumor activity. To experimentally test this hypothesis, we performed CD19‐CAR T cell killing assays in vitro in the presence or absence of tacrolimus or everolimus. To identify a suitable target to conduct such experiments we first screened a panel of four CD19‐expressing DLBCL cell lines, namely, Farage, DOHH‐2, SUDHL‐10 and OCI‐LY‐8, for their sensitivity to tacrolimus and everolimus in vitro. All four lymphoma cell lines showed sensitivity to everolimus (Figure S1), making them unsuitable targets. To avoid interference of mTOR inhibition on tumor growth, we selected the acute myeloid leukemia cell line OCI‐AML2 which we retrovirally engineered to overexpress CD19 at its surface (Figure S2A,B). After confirming that it was not sensitive to tacrolimus or everolimus exposure (Figure S2C), we employed the OCI‐AML2‐CD19 cell line as target to evaluate the effect of tacrolimus and everolimus on CAR T‐cell activity. OCI‐AML2‐CD19 cells were co‐cultured with CD19.CD28z CAR T cells at an effector‐to‐target (E:T) ratio of 2:1 in the presence or absence of tacrolimus and everolimus over a 7‐days in vitro killing assay. CAR T cells failed to control tumor growth in the presence of tacrolimus at both 5 and 10 ng/ml (Figure 2A,B). Conversely, CAR T cells efficiently controlled tumor growth in the presence of everolimus at doses as high as 10 ng/mL, at an extent similar to what observed in the DMSO control conditions (Figure 2A,B). Collectively, these in vitro results support our hypothesis that mTOR inhibition by everolimus represents a permissive IST option allowing CAR T‐cell antitumor activity while tacrolimus exposure completely blocked it.

**FIGURE 2:**
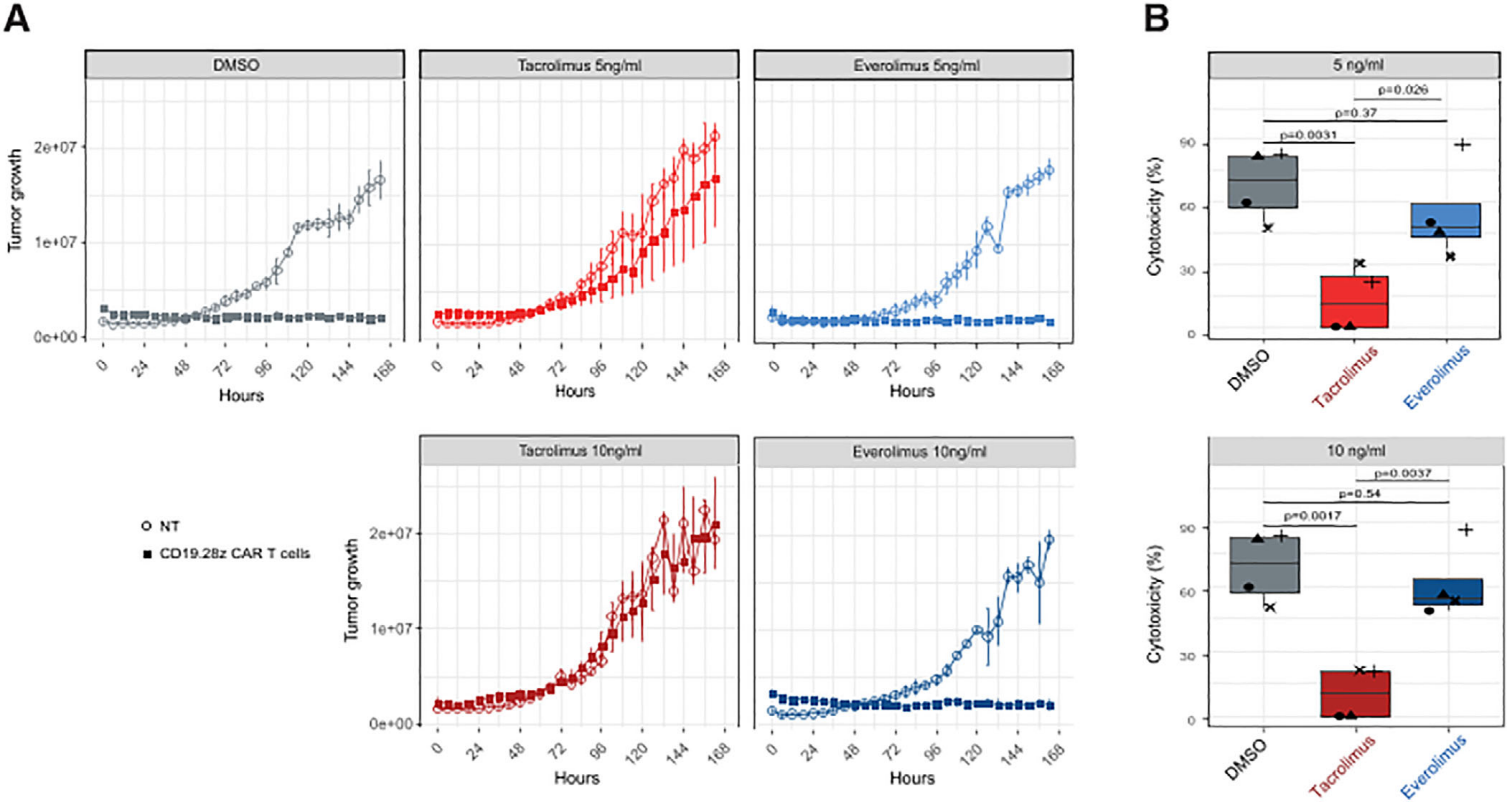
Everolimus but not tacrolimus allows CAR T cell cytotoxicity in vitro. (A) Tumor growth of OCI‐AML2‐CD19 in the presence (square marked curve) or absence (open circles) of CD19 CAR T cells at an E:T ratio 2:1, in the presence of tacrolimus at 5 ng/mL (light red), 10 ng/mL (dark red), and everolimus 5 ng/mL (light blue) and 10 ng/mL (dark blue), compared to DMSO (grey). Tumor growth was monitored using Incucyte live cell imagining over 7 days. (B) Cytotoxic effect of CD19 CAR T cells derived from different healthy donors on OCI‐AML2‐CD19 cells 6 days after treatment with tacrolimus and everolimus at 5 (upper panel) or 10 (lower panel) ng/mL.

## 4 Discussion

This report contributes to the growing body of evidence that CAR T‐cell therapy can be safely and efficiently administered to SOT recipients with relapsed/refractory PTLD. Moreover, it integrates preclinical validation of tacolimus and everolimus effects on CAR T‐cell activity thus providing elements to guide the management of IST after CAR T cells. In addition to everolimus, other immunosuppressive agents may preserve CAR T‐cell function, particularly the mechanistically related mTOR inhibitor sirolimus (rapamycin). This possibility remains to be investigated and may be especially relevant in cases of everolimus intolerance or toxicity. Translating these insights into clinical care, our patient achieved durable remission with only low‐grade toxicity, despite the complex immunologic terrain of SPK.

In summary, we describe a challenging case of relapsing/refractory PTLD complicated with acute graft rejection at time of leukapheresis who achieved CR after axicabtagene ciloleucel infusion during CNI‐free, mTOR‐based immunosuppression. The treatment was well tolerated, and we observed no new episodes of graft rejection after CAR T cell infusion. Our in vitro data indicate that everolimus but not tacrolimus is permissive to CAR T‐cell mediated killing. Collectively, our data support the use of mTOR‐based, CNI‐free immunosuppression during CAR T‐cell therapy of hematological malignancies in SOT recipients.

## Author Contributions

MM was responsible for patient management, data collection, and manuscript drafting. YHY and SW were responsible for in vitro evaluation, data collection, and manuscript drafting. FS supervised the project and reviewed the final manuscript. CDRO, SM, SML, AP, AM, PP, CB, LH, KE, JD, TL, FB, YC, FH, SdS, JV, VO read and approved the final version of the manuscript.

## Funding

This work was partly supported by grants from the Swiss National Science Foundation (310030_220070/1), the Fondation Privée des HUG (RS09‐08), the Peter Anton & Anna Katharina Miescher, the Henriette Meyer Foundation, and the Dr Henri Dubois‐Ferrière Dinu Lipatti Foundation.

## Ethics Statement

Ethical review and approval were not required for this case report in accordance with local legislation and institutional requirements. The report was prepared in accordance with the ethical principles outlined in the Declaration of Helsinki.

## Consent

The patient provided her written informed consent to participate in this study.

## Conflicts of Interest

Federico Simonetta: institutional consulting fees from BMS/Celgene, Incyte, Kite/Gilead; speaker fees from Kite/Gilead, Incyte; travel support from Kite/Gilead, Novartis, AstraZeneca, Neovii, Janssen; research funding from Kite/Gilead, Novartis, BMS/Celgene. Chiara Bernardi: Research funding from BMS/Celgene Yves Chalandon: institutional consulting fees for advisory board from MSD, Novartis, Incyte, BMS, Pfizer, Abbvie, Roche, Jazz, Gilead, Amgen, Astra‐Zeneca, Servier, Takeda, Pierre Fabre, Medac; travel support from MSD, Roche, Novartis, Pfizer, BMS, Gilead, Amgen, Incyte, Abbvie, Janssen, Astra‐Zeneca, Jazz, Pierre Fabre, Sanofi all via the institution.

## Supporting information




**Supplemental Figure 1**: Effect of tacrolimus and everolimus on tumor growth in four B cell lymphoma cell lines. Tumor growth of four lymphoma cell lines (Farage, DOHH‐2, SUDHL‐10, and OCI‐LY‐8) following treatment with DMSO control (grey), tacrolimus at 5 ng/ml (light red) and 10 ng/ml (dark red), everolimus at 5 ng/ml (light blue) and 10 ng/ml (dark blue).

**Supplemental Figure 2**: OCI‐AML2‐CD19 cells show resistance to tacrolimus and everolimus. (A) Plasmid map of the CD19 construct used to generate the engineered OCI‐AML2‐CD19 cell line. (B) Flow cytometry analysis confirming overexpression of CD19 in the modified cell line. (C) OCI‐AML2‐CD19 tumor growth after treatment with DMSO (grey), tacrolimus at 5 ng/mL (light red) and 10 ng/mL (dark red), and everolimus at 5 ng/mL (light blue) and 10 ng/mL (dark blue).

## Data Availability

The data supporting the findings of this report are contained within the article. Additional information may be available from the corresponding author upon reasonable request.

## References

[1] M. Markouli , F. Ullah , and N. Omar , “Recent Advances in Adult Post‐Transplant Lymphoproliferative Disorder,” Cancers 14, no. 23 (2022): 5949, 10.3390/cancers14235949. PMC9740763 36497432

[2] S. S. Neelapu , F. L. Locke , and N. L. Bartlett , Axicabtagene Ciloleucel CAR T‐Cell Therapy in Refractory Large B‐Cell Lymphoma (New England Journal of Medicine, 2017), 2531–2544. 10.1056/NEJMoa1707447 PMC5882485 29226797

[3] S. J. Schuster , M. R. Bishop , and C. S. Tam , Tisagenlecleucel in Adult Relapsed or Refractory Diffuse Large B‐Cell Lymphoma (New England Journal of Medicine, 2019), 45–56. 10.1056/NEJMoa1804980 30501490

[4] U. Ibrahim , C. Bassil , and J. C. Chavez , “CAR T Cell Therapy for Post‐Transplant Lymphoproliferative Disorder After Solid Organ Transplantation: A Safe and Feasible Therapy for an Orphan Disease,” Clinical Lymphoma Myeloma and Leukemia 23, no. 10 (2023): 772–778, 10.1016/j.clml.2023.05.007. 37263876

[5] S. Krishnamoorthy , A. Ghobadi , and R. D. Santos , “CAR‐T Therapy in Solid Organ Transplant Recipients With Treatment Refractory Posttransplant Lymphoproliferative Disorder,” American Journal of Transplantation 21, no. 2 (2021): 809–814, 10.1111/ajt.16367. 33089906

[6] E. Luttwak , D. Hagin , and C. Perry , “Anti‐CD19 CAR‐T Therapy for EBV‐Negative Posttransplantation Lymphoproliferative Disease—A Single Center Case Series,” Bone Marrow Transplantation 56, no. 5 (2021): 1031–1037, 10.1038/s41409-020-01145-1. 33230187

[7] O. Mamlouk , R. Nair , and S. P. Iyer , “Safety of CAR T‐Cell Therapy in Kidney Transplant Recipients,” Blood 137, no. 18 (2021): 2558–2562, 10.1182/blood.2020008759. PMC8109014 33512386

[8] M. McKenna , N. Epperla , and A. Ghobadi , “Real‐World Evidence of the Safety and Survival With CD19 CAR‐T Cell Therapy for Relapsed/Refractory Solid Organ Transplant‐Related PTLD,” British Journal of Haematology 202, no. 2 (2023): 248–255, 10.1111/bjh.18828. 37129856

[9] A. Panopoulou , V. Mehra , and K. Cwynarski , “CAR‐T Cell Therapy for PTLD: Analysis of CAR‐T Product, Engraftment, and Outcomes in Patients Receiving Parallel Immunosuppression,” EJHaem 6, no. 1 (2025): e70006, 10.1002/jha2.70006. PMC11840709 39981112

[10] R. Alaggio , C. Amador , and I. Anagnostopoulos , “The 5th Edition of the World Health Organization Classification of Haematolymphoid Tumours: Lymphoid Neoplasms,” Leukemia 36, no. 7 (2022): 1720–1748, 10.1038/s41375-022-01620-2. PMC9214472 35732829

[11] R. U. Trappe , D. Dierickx , and H. Zimmermann , “Response to Rituximab Induction Is a Predictive Marker in B‐Cell Post‐Transplant Lymphoproliferative Disorder and Allows Successful Stratification Into Rituximab or R‐CHOP Consolidation in an International, Prospective, Multicenter Phase II Trial,” Journal of Clinical Oncology 35, no. 5 (2017): 536–543, 10.1200/JCO.2016.69.3564. 27992268

[12] S. P. Berger , C. Sommerer , and O. Witzke , “Two‐Year Outcomes in De Novo Renal Transplant Recipients Receiving Everolimus‐Facilitated Calcineurin Inhibitor Reduction Regimen From the TRANSFORM Study,” American Journal of Transplantation 19, no. 11 (2019): 3018–3034, 10.1111/ajt.15480. 31152476

